# Serum-Mediated Cleavage of *Bacillus anthracis* Protective Antigen Is a Two-Step Process That Involves a Serum Carboxypeptidase

**DOI:** 10.1128/mSphere.00091-18

**Published:** 2018-06-27

**Authors:** David L. Goldman, Edward Nieves, Antonio Nakouzi, Johanna Rivera, Ei Ei Phyu, Than Htut Win, Jacqueline M. Achkar, Arturo Casadevall

**Affiliations:** a Department of Microbiology and Immunology, Einstein College of Medicine, Bronx, New York, USA; b Department of Pediatrics, Einstein College of Medicine, Bronx, New York, USA; c Department of Biochemistry, Einstein College of Medicine, Bronx, New York, USA; d Department of Medicine at the Albert Einstein College of Medicine, Bronx, New York, USA; e The Children’s Hospital at Montefiore, Bronx, New York, USA; University of Texas Health Science Center

**Keywords:** anthrax, proteases, toxin

## Abstract

Much of our understanding of the activity of anthrax toxin is based on *in vitro* systems, which delineate the interaction between Bacillus anthracis toxins and the cell surface. However, these systems fail to account for the intimate association of B. anthracis with the circulatory system, including the contribution of serum proteins to the host response and processing of anthrax toxins. Using a variety of immunological techniques to inhibit serum processing of B. anthracis protective antigen (PA) along with mass spectrometry analysis, we demonstrate that serum digests PA via 2 distinct reactions. In the first reaction, serum cleaves PA_83_ into 2 fragments to produce PA_63_ and PA_20_ fragments, similarly to that observed following furin digestion. This is followed by carboxypeptidase-mediated removal of the carboxy-terminal arginine and lysines from PA_20_.

**IMPORTANCE** Our findings identify a serum-mediated modification of PA_20_ that has not been previously described. These observations further imply that the processing of PA is more complex than currently thought. Additional study is needed to define the contribution of serum processing of PA to the host response and individual susceptibility to anthrax.

## Abstract (precis)

Our findings identify a serum-mediated modification of PA_20_ that has not been previously described. These observations further imply that the processing of PA is more complex than currently thought. Additional study is needed to define the contribution of serum processing of PA to the host response and individual susceptibility to anthrax.

## INTRODUCTION

Bacillus anthracis is the causative agent of anthrax and is widely recognized for its potential use as an agent of bioterrorism. B. anthracis secretes 2 bipartite toxins, the lethal toxin and the edema toxin, that are essential for virulence. Both toxins require the protective antigen (PA) component to mediate cell entry. PA is, therefore, essential to the damaging effects of anthrax toxins, and PA-deficient mutants are avirulent (1).

The current paradigm of toxin pathogenesis posits that B. anthracis secretes the proform of PA (PA_83_), which binds to cell surface receptors (tumor endothelium marker-8 or capillary morphogenesis protein-2), where it undergoes cleavage by cell-associated furin into 2 fragments, PA_20_ and PA_63_. PA_63_ subsequently undergoes heptamerization to form a prepore structure that binds edema factor (EF) or lethal factor (LF) and is internalized. Understanding the mechanism by which anthrax toxin is processed is important because interference with the processing steps is the basis for the development of therapeutics, including furin inhibitors (2). In addition, antibodies (Abs) reactive to PA are protective in animal models of anthrax and one monoclonal antibody, raxibacumab, has been licensed for clinical use (3–5).

Much of our understanding about toxin processing in anthrax pathogenesis is based on experiments using *in vitro* systems (reviewed in reference 6). Nonetheless, these models fail to take into account the role of host serum proteins as part of the host response to anthrax. During the course of anthrax, B. anthracis encounters serum proteins at multiple stages, including invasion of the lymphatic system and high-level bacteremia, which occurs in the context of sepsis. In late stages of experimental anthrax in macaques, for example, lethal toxin concentrations on the order of 10 µg/ml have been reported (7). The intimate association between B. anthracis and serum is further highlighted by the presence of pathogen-associated proteins that directly act on elements within the circulation. This includes enzymes that digest host hemoglobin and circulating lethal toxin, which interferes with neutrophil function (8, 9).

Several lines of evidence suggest that PA processing is more complex than is apparent from the current model. Anthrax toxin is released from B. anthracis in vesicles that contain all toxin components (10). Although these vesicles may be rapidly disrupted by serum albumin-releasing toxin components (11), they are also released intracellularly. In addition, PA circulating in the serum is found in animal models as a complex of PA_63_ bound to LF or EF rather than as intact PA_83_ (12). In fact, serum from humans and other species has been shown to contain proteolytic activity that digests PA in a manner similar to that seen with furin (13–15). Our previous studies suggest a correlation between serum-mediated digestion of PA and protection from the killing effects of lethal toxin *in vitro* (15). In the current work, we found that serum-mediated processing of PA is a 2-step reaction that involves carboxypeptidase (CP)-mediated truncation of the PA_20_ fragment.

## RESULTS

### Serum-mediated digestion of rPA.

**FIG 1:**
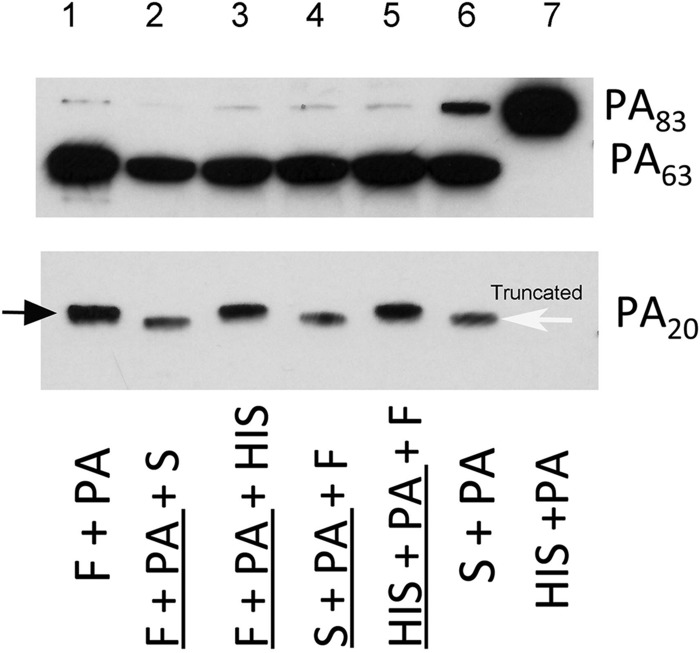
Serum-mediated digestion of rPA83 produces a truncated PA_20_ fragment compared with furin-mediated digestion. Shown are the digestion fragments of PA_83_ under conditions of incubation with furin (F; lane 1), serum (S; lane 6), or heat-inactivated serum (HIS; lane 7). Treatment of rPA83 with serum after and prior to furin digestion (lanes 2 and 4, respectively) produced a truncated PA_20_ fragment, indicating that truncation is distinct from furin digestion. In contrast, incubation of furin-treated PA_83_ with HIS (lanes 3 and 5) failed to result in PA_20_ truncation, suggesting that this process is heat labile. For the purpose of the assay, serum was incubated with PA_63_ for 1 h. MAb 10F4 (which recognizes domains 2 and 4 of PA_83_) was used to detect the PA_63_ fragment, while MAb 19D9 (which recognizes domain 1) was used to detect both the normal and truncated PA_20_ fragments. The black arrow points to the normal PA_20_ fragment, while the white arrow points to the truncated PA_20_ fragment. This experiment was done 2 times with similar results. Underlining indicates preincubation.

Serum treatment of recombinant PA_83_ (rPA_83_) produced 2 protein fragments, PA_63_ and a band that is slightly lower in molecular mass than PA_20_ (Fig. 1; lane 6). The larger protein is similar in size to the PA_63_ protein produced by furin digestion of rPA_83_. However, the smaller protein is smaller than the PA_20_ protein produced by furin digestion of rPA_83_ and is referred to as truncated PA_20_. Furthermore, serum treatment of rPA83 before or after furin digestion still produced this truncated fragment (Fig. 1, lanes 2 and 4). Heat inactivation of serum prevented this truncation (Fig. 1; lanes 3 and 5), consistent with the idea that the enzyme responsible for truncation is heat labile.

### Inhibition of serum-mediated digestion of rPA.

To determine the precise site at which serum cleaves rPA, we attempted to inhibit serum-mediated cleavage using a library of overlapping peptides, which represent the PA sequence and antibodies that recognize various PA sites. Preincubation of rPA with the 19D2 monoclonal antibody (MAb), which recognizes an epitope immediately C terminal of the furin site (16), prevented rPA digestion by serum and furin. This inhibition of digestion was not seen with other PA-specific antibodies, including 7.5G, which recognizes domain 1 of PA_83_. Serum-mediated PA cleavage was also prevented by coincubation of serum with 3 overlapping peptides (D5, D6, and D7), which contain the furin digestion site, but not with other peptides (including D12, E1, and E2, which represent PA sequences approximately 30 amino acid [AA] residues C terminal to the furin site) (not shown).

**FIG 2:**
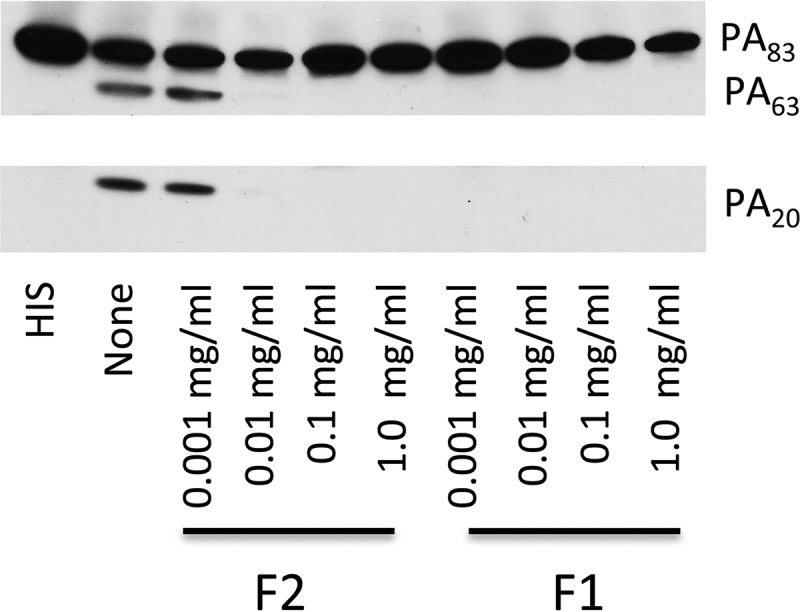
Furin inhibitors I and II prevent serum-mediated digestion of rPA_83_. Heat-inactivated serum (HIS) had no effect on rPA. In the absence of inhibitor (none), PA_83_, PA_63_, and PA_20_-like fragments were present. Both furin inhibitor I and furin inhibitor II (F1 and F2) prevented serum digestion of PA_83_. PA_83_ was incubated for the serum for 30 min. This experiment was repeated 3 times with similar results.

Using chemical inhibitors while measuring PA_63_ formation, we found that the serine/cysteine protease antipain partially inhibited the formation of PA_63_. In contrast, none of the other tested protease inhibitors, including bestatin, chymostatin, E-64, leupeptin, pepstatin, phosphoramidon, Pefabloc SC, and aprotinin, prevented PA_63_ formation. As in previous studies, we found that EDTA was a potent inhibitor of serum-mediated digestion of PA_83_. In contrast, both competitive inhibitors of furin (I and II) prevented serum-mediated digestion of PA. For furin inhibitor I, concentrations as low as 0.001 mg/ml resulted in complete inhibition of serum digestion, whereas for furin inhibitor II, concentrations as low as 0.010 mg/ml produced complete inhibition of digestion (Fig. 2).

### Truncated PA_20_ fragment.

**FIG 3:**
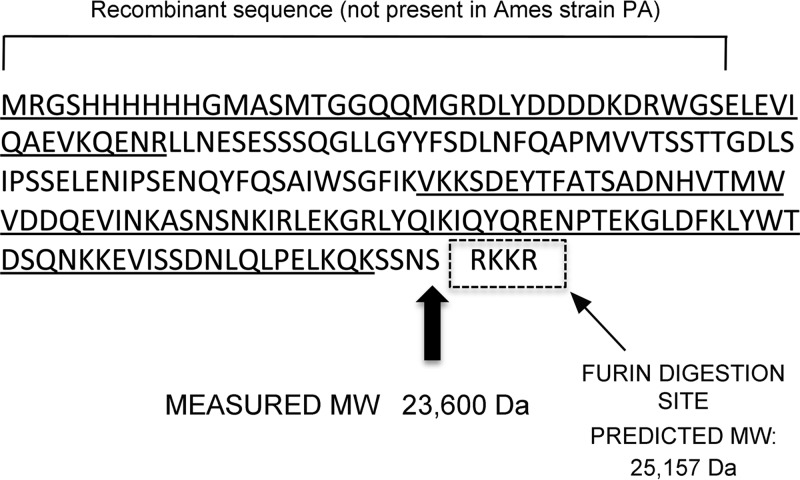
Mass spectrometry of the serum-truncated PA_20_ fragment. The predicted size of the fragment to the SNSS amino acid sequence is 23,588 Da (thick arrow), a difference of 12 Da or 0.06% compared with the measured mass of 23,600 Da. In contrast, digestion at the furin consensus site should produce a PA_20_ fragment with a mass of 25,157 Da. Underlined peptide sequences were detected by MS analysis. The dotted box represents the consensus recognition site for furin.

To better identify the precise site of serum-mediated digestion of rPA, the truncated PA_20_ fragment produced by serum digestion was examined by mass spectrometry (MS). First, the intact-protein mass of this fragment was measured and the experimental mass determined by liquid chromatography-electrospray ionization mass spectrometry (LC-ESI MS) to be 23,600 Da (Fig. S1). Furin cleaves at RXK/RR, which would correspond to a predicted molecular mass of 25,157 Da for rPA (Fig. 3; N terminus to RKKR), which represents a difference of 1,557 Da (a value far beyond the error of measurement). To determine the sequence of the truncated PA_20_ fragment, in-gel trypsin digestion was performed. The liquid chromatography-tandem mass spectrometry (LC-MS/MS) data identified the underlined tryptic peptides shown in Fig. 3 (identified peptides from this tryptic digest are listed in Table S1). The peptide sequence LLNES … GFIK is too large for fragmentation on an LTQ (linear ion trap quadrupole) mass spectrometer and was not detected by MS/MS, but the + 4, +5, +6, +7, and +8 charge states were detected (Fig. S2). The predicted protein mass from the N terminus to the last tryptic peptide identified is 23,213 Da and if the next 4 amino acids (SSNS) are included the predicted protein mass increases to 23,588 Da, a difference of 12 Da or 0.05% compared with the experimental intact-protein mass (23,600 Da). These findings are consistent with serum-mediated cleavage of the basic, C-terminal arginine and lysine residues from the PA_20_ fragment produced by furin digestion, possibly followed by carboxypeptidase.

10.1128/mSphere.00091-18.1 FIG S1  Deconvoluted experimental mass for the truncated PA20 fragment obtained from the intact-protein LC-MS measurement. Download FIG S1, TIF file, 1.2 MB. Copyright © 2018 Goldman et al. 2018 Goldman et al. This content is distributed under the terms of the Creative Commons Attribution 4.0 International license.

10.1128/mSphere.00091-18.2 FIG S2  Extraction ion chromatogram (I) of the +4, +5, +6, +7, and +8 charge states (A to E) of the peptides from rPA83 following trypsin digestion minus LLNESESSSQGLLGYYFSDLNFQAPMVVTSSTTGDLSIPSSELENIPSENQYFQSAIWSGFIK. The mass spectra for the 45.6-min retention time peak corresponding to this peptide is shown (II). Download FIG S2, TIF file, 1.5 MB. Copyright © 2018 Goldman et al. 2018 Goldman et al. This content is distributed under the terms of the Creative Commons Attribution 4.0 International license.

10.1128/mSphere.00091-18.3 TABLE S1  Peptides identified following trypsin digestion of truncated PA_20_ protein. Download TABLE S1, TIF file, 1.8 MB. Copyright © 2018 Goldman et al. 2018 Goldman et al. This content is distributed under the terms of the Creative Commons Attribution 4.0 International license.

### Carboxypeptidase treatment of rPA.

**FIG 4:**
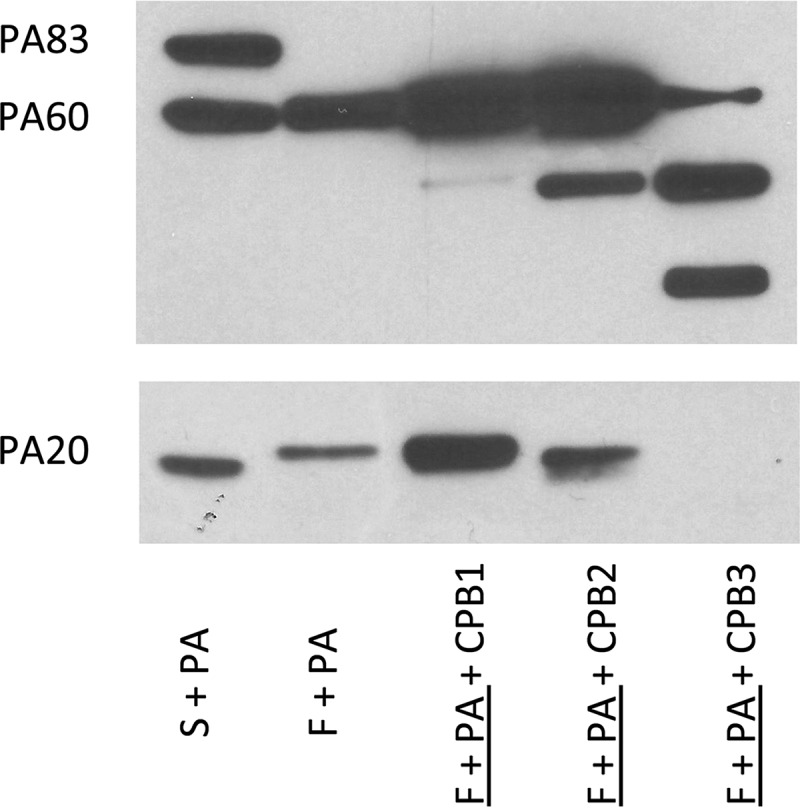
Carboxypeptidase B (CPB) treatment of furin-digested PA produces a truncated PA_20_ fragment. Treatment of furin-digested rPA_83_ with CPB from pig pancreas resulted in a dose-related truncation of the PA_20_ fragment. This was most apparent for CPB2 (25 µg/ml) compared to lower concentrations of CPB1 (2.5 µg/ml). Incubation with higher CPB3 concentrations (250 µg/ml) resulted in complete loss of PA_20_ reactivity and the appearance of multiple digestion fragments. Underlining indicates preincubation.

Given these results, we sought to determine whether this truncated PA_20_ fragment could result from serum carboxypeptidase digestion of PA_20_. Carboxypeptidases are a family of enzymes that cleave residues from the C-terminal end of a protein. This includes a group of enzymes that cleave basic amino acid residues from the carboxy terminus. To determine if carboxypeptidase could produce a truncated PA_20_ fragment, we conducted studies with a pancreatic carboxypeptidase. The effects of carboxypeptidase B (CPB) treatment on furin-digested rPA were dose dependent. At higher concentrations (250 µg/ml) (Fig. 4, lane 5), multiple digestion fragments of PA were observed and PA_20_ reactivity was completely lost. A similar pattern was seen in the absence of furin and presumably relates to the presence of contaminating trypsin in this pancreatic preparation. In contrast, at lower concentrations of CPB (25 µg/ml) (Fig. 4, lane 4), treatment produced a truncated PA_20_ fragment that was similar in size to that observed with serum digestion of PA (Fig. 4, lane 1). Lower concentrations of CPB (2.5 µg/ml) had no effect on the size of furin-treated PA_20_ compared with the results seen with furin treatment alone.

### Inhibition of serum carboxypeptidase activity.

**FIG 5:**
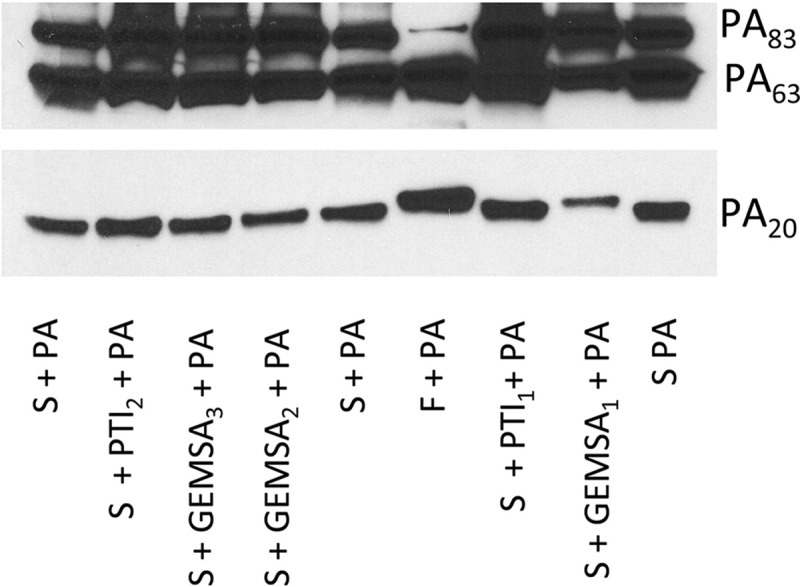
GEMSA, but not PTI, prevented truncation of PA_20_. In the presence of high concentrations of GEMSA (GEMSA1; 500 µg/ml), inhibition of serum-mediated truncation of PA_20_ was present. In contrast, PTI at concentrations as high as 1.25 mg/ml (PTI1) failed to inhibit serum-mediated truncation of PA_20_. Lower concentrations of GEMSA (50 and 5 µg/ml; GEMSA2 and GEMSA3) and PTI (PTI2; 125 µg/ml) did not prevent serum-mediated truncation of PA_20_. This experiment was done 3 times with similar results.

Next, we sought to determine whether the ability of serum to produce a truncated PA_20_ fragment could be inhibited by carboxypeptidase inhibitors. Both guanidinoethylmercaptosuccinic acid (GEMSA) and potato tuber extract (PTI) are potent competitive inhibitors of carboxypeptidase, though their inhibitory activity is not specific to any one class of carboxypeptidases. Addition of GEMSA (500 µg/ml) to serum prevented the formation of a truncated PA_20_ and resulted in a PA_20_ fragment that was more similar in size to that produced by furin digestion (Fig. 5). In contrast, no inhibition was seen with lower concentrations of GEMSA and for all concentrations of carboxypeptidase inhibitor (PTI) from potato tuber extract.

## DISCUSSION

B. anthracis and the toxins that it secretes have an intimate association with the circulation and with serum over the course of infection. Our studies confirm earlier reports that both human and animal sera contain a furin-like enzyme, which digests PA to produce PA_63_ and PA_20_ fragments (13–15). In our own studies, this activity was associated with protection against lethal toxin *in vitro* (15). We now extend these findings to demonstrate that human serum contains a carboxypeptidase which further processes the PA_20_ fragment by removing the C-terminal basic amino acid residues, resulting in a truncated PA_20_ fragment. These findings contrast with the current model of anthrax toxin, which suggests that processing of PA occurs only at the cell surface, and provide additional evidence for the complexity of anthrax toxin mechanisms of action. However, we note that serum and cell surface PA processing are not mutually exclusive events.

PA_20_ has been detected in the blood of B. anthracis-infected animals, though its contribution to anthrax pathogenesis is unknown (17). Nonetheless, several lines of evidence suggest that it may play an active role in infection. For example, PA_20_ contains a PA_14_ domain that is conserved among bacterial toxins and appears to play a role in cell binding (18). Hammamieh et al. reported that exposure of human peripheral blood mononuclear cells to PA_20_ induced a variety of genes related to the inflammatory system and to cell migration and triggered apoptosis in these cells (17). Furthermore, PA_20_ has been reported to bind lethal factor (19). Although circumstantial, these findings are consistent with a role for PA_20_ in the pathogenesis of anthrax.

Serum is known to contain 2 carboxypeptidases, CP-N and CPB_2_ (which is also known as CPU, plasma carboxypeptidase B, and thrombin-activatable fibrinolysis inhibitor). Both carboxypeptidases cleave carboxy-terminal arginine and lysine residues from peptides/proteins and have been implicated in regulating inflammation through their effects on serum protein cascades, such as the complement anaphylatoxins and kinins (20). As members of the carboxypeptidase family, both CP-N and CPB_2_ contain a zinc-binding site that makes them susceptible to inhibition by metal chelators. CP-N is constitutively produced by the liver, with serum concentrations on the order of 30 µg/ml (21). In contrast, CPB_2_ must be activated by fibrin and, once activated, downregulates fibrinolysis by removing terminal lysines from fibrin and is present in serum concentrations on the order of 4 to 15.0 µg/ml (22, 23). Elevated levels of CPB_2_ have been found both in animal models of bacterial sepsis and in septic patients and have been hypothesized to play a role in the hypercoagulability associated with sepsis (24–26). Interestingly, both carboxypeptidases have been shown to inactivate complement anaphylatoxins (27, 28). Furthermore, both C3 and C5 have been implicated in the host response to anthrax (29, 30). Thus, PA_20_ may possibly alter anthrax pathogenesis by interfering with anaphylatoxin inactivation during anthrax-associated sepsis.

It is interesting that CP-N is more susceptible to inhibition by GEMSA whereas CPB_2_ is more susceptible to inhibition by potato carboxypeptidase inhibitor (31). Thus, our findings are consistent with the hypothesis that *in vitro*, CP-N is primarily responsible for the observed truncation of PA_20_. Nonetheless, the precise carboxypeptidase responsible for the truncation of PA_20_ *in vivo* (including during the sepsis of anthrax) is not known and it is likely that there is redundancy to the process. Of note, macrophages also express a membrane-associated carboxypeptidase (CP-M) that cleaves C-terminal lysines and arginine residues from proteins (32). It is, therefore, likely that a similar form of processing occurs at the surface of target cells.

In summary, we demonstrate that serum processing of PA is a 2-step process that involves a furin-like digestion of the PA_83_ component followed by truncation of the PA_20_ fragment by serum carboxypeptidases. The significance of these 2 serum-associated activities remains to be defined. On the basis of earlier studies that associated furin-like digestion with protection, we believe that this activity may in fact contribute to the host response to anthrax. This would be consistent with the close association of B. anthracis with the circulatory system. We also suggest that it is possible that the variations in these serum proteolytic activities contribute to differences in individual susceptibilities to anthrax. Additional studies examining gain and loss of function in the context of experimental infection may help further delineate the importance of these processes.

## MATERIALS AND METHODS

### PA.

Recombinant PA_83_ (rPA) and its amino acid sequence were obtained from Wadsworth Laboratories, New York State Department of Health (Albany, NY).

### Sera.

Serum was obtained from laboratory volunteers and stored at −80°C with approval from the Committee of Clinical Investigations at Albert Einstein College of Medicine. In some experiments, pooled sera, processed to retain complement activity (Sigma, St. Louis, MO), was used. These commercial sera produced results comparable to those obtained with sera from human volunteers.

### Antibodies and peptides.

A library of 6 murine monoclonal antibodies (7.5G, 16A12, 10F4, 19D9, 20G7, and 2H9) that were previously generated and characterized was used both to define the digestion site and as detection reagents for immunoblot studies (33). Binding sites for these antibodies are provided in Table S2. A previously synthesized library of overlapping peptides which represents the PA sequence was used for inhibition studies (16).

10.1128/mSphere.00091-18.4 TABLE S2  PA binding domains of various MAbs used in this study. Download TABLE S2, DOCX file, 0.04 MB. Copyright © 2018 Goldman et al. 2018 Goldman et al. This content is distributed under the terms of the Creative Commons Attribution 4.0 International license.

### Proteolytic digestion and fragment detection.

Proteolytic digestion studies were performed as previously described (15). Briefly, rPA (2.5 µg) was incubated with 25 µl of serum, phosphate-buffered saline, or furin (Invitrogen) (0.5 units) at 37°C for 30 to 60 min. In some experiments, serum was heat treated at 56°C for 30 min prior to incubation with toxin. In other experiments, protease inhibitors (see below) or peptides at a concentration of 5 µg/ml were added to serum prior to incubation with rPA. Digested rPA was separated by SDS-electrophoresis and transferred to a nitrocellulose membrane. Membranes were blocked with 5% milk and then incubated with primary antibody. The following MAbs were used to characterize rPA cleavage: 10F4 (IgG1) and 7.5G (IgG2b). All MAbs were used at a concentration of 0.25 µg/ml. Primary antibody was detected with horseradish peroxidase-labeled goat isotype-specific antibody at a dilution of 1:25,000. Proteins were visualized by development with an ECL chemiluminescence kit (Pierce, Rockford, IL).

### Inhibition studies. (i) Peptides.

Serum (24 µl) was incubated with individual biotinylated peptides, peptide mixtures, or phosphate-buffered saline (PBS) for 2 h at room temperature. These peptides were chosen from a library of peptides representing the entire length of rPA and were synthesized as 15-mer, overlapping by 10 residues (16). This serum peptide mixture was then incubated with 1.5 µg of rPA for 30 min at 37°C, and the resulting mixture was subjected to separation by SDS-PAGE and detection by Western blotting.

### (ii) MAbs.

PA (1.5 µg) was incubated with one of several PA-specific MAbs (2 µg) (33) for 10 min at room temperature. This mixture was then added to 24 µl of serum, incubated at 37°C for 20 min, and then subjected to SDS-electrophoresis and immunoblotting.

### (iii) Protease inhibitors.

A volume of 10 µl of sera was preincubated with 1 of 9 protease inhibitors included in a commercially available protease inhibitor set (Roche) for 30 min at 30°C. Individual inhibitors (including antipain, bestatin, chymostatin, E-64, phosphoramidon, Pefabloc SC, and aprotinin) were reconstituted per the instructions of each manufacturer. Following this incubation, 1.5 µg of rPA was added to the mixture and incubated at 37°C for 1 h. Specific inhibition of furin activity was accomplished using furin inhibitor I (Cayman Chemical Company) and furin inhibitor II (Sigma). These compounds are selective competitive inhibitors of the proprotein convertases, including furin. Serum (12 µl) was incubated with furin inhibitors at room temperature for 10 min, after which rPA (1.5 µg) was added and the entire mixture incubated for an additional 1 h at 37°C.

### (iv) Carboxypeptidase inhibition.

For these experiments, sera were pretreated with a variety of inhibitors for 30 min prior to incubation with rPA. These inhibitors included the following: guanidinoethylmercaptosuccinic acid (GEMSA) (Santa Cruz Biotechnology) and carboxypeptidase inhibitor from potato tuber extract (Sigma). The serum-PA digestion mixture was separated by electrophoresis. PA_63_-like and truncated PA_20_ fragments were then detected with antibodies 10F4 and 19D2, respectively.

### Mass spectrometry (MS).

To isolate the truncated PA_20_ molecule, serum-digested rPA was incubated overnight at 4°C with 200 µl of protein G resin in binding buffer (20 mM Tris, 150 mM NaCl, pH 7.4) together with 50 µg of MAb 19D2. The resultant slurry was centrifuged for 2.5 min at 2,500 × *g* and the resin washed 5 times with binding buffer (Pierce). Following elution, the protein was separated in a nondenaturing gel and electroeluted for further analysis.

Mass spectrometry (MS) measurements and liquid chromatography (LC) separations were obtained on an LTQ (linear ion trap quadrupole) mass spectrometer (Thermo Scientific, San Jose, CA), an LC 3000 rapid-separation system (Dionex Corporation, Sunnyvale, CA) was used for processing of tryptic peptides, and an HP Agilent 1100 series system was used for intact-protein separation. For intact-protein molecular weight measurements of the electro-eluted protein, a C4 Vydac TP column (1 by 50 mm; 300 Å; 50 µl/min) was used. After desalting performed with 1% acetonitrile–0.1% aqueous formic acid (FA) for 2 min, the protein was eluted after increasing the level of acetonitrile to 55% acetonitrile–0.1% aqueous FA. The mass range from 600 to 1,800 *m*/*z* was acquired on the LTQ mass spectrometer, and the raw data were deconvoluted using MagTran (34) or ProMass (Thermo Fisher Scientific). Another aliquot of the electroeluted protein was separated on a one-dimensional (1D) SDS gel, and selected molecular weight bands were excised for in-gel tryptic digestion as described previously (35). After sample injection and LC peptide separation (using an acetonitrile gradient), the 10 most abundant ions obtained from the survey scan (300 to 1,600 m/*z*) were selected for fragmentation (MS/MS). Normalized collision energy of 35% and a 2 *m*/*z* isolation width were used for MS/MS. The MS/MS data were converted to a text file for peptide/protein identification using Mascot (Matrix Science, Inc.).

### Carboxypeptidase-mediated digestion of PA.

To determine whether carboxypeptidase digestion of furin-treated rPA could produce a fragment similar in size to that seen with serum digestion of rPA, experiments were done with carboxypeptidase B (CPB) (Sigma). For these experiments, rPA was treated with furin for 10 min at 30°C and the mixture was incubated with CPB at different concentrations at 37°C. Proteins were separated by SDS-PAGE and detected by immunoblotting as described above.
