# Supplementary figures and images for: Serum-Mediated Cleavage of Bacillus anthracis Protective Antigen Is a Two-Step Process That Involves a Serum Carboxypeptidase
Source: mSphere. 2018 Jun 27;3(3):e00091-18. doi: 10.1128/mSphere.00091-18 (PMC6021598; doi:10.1128/mSphere.00091-18)

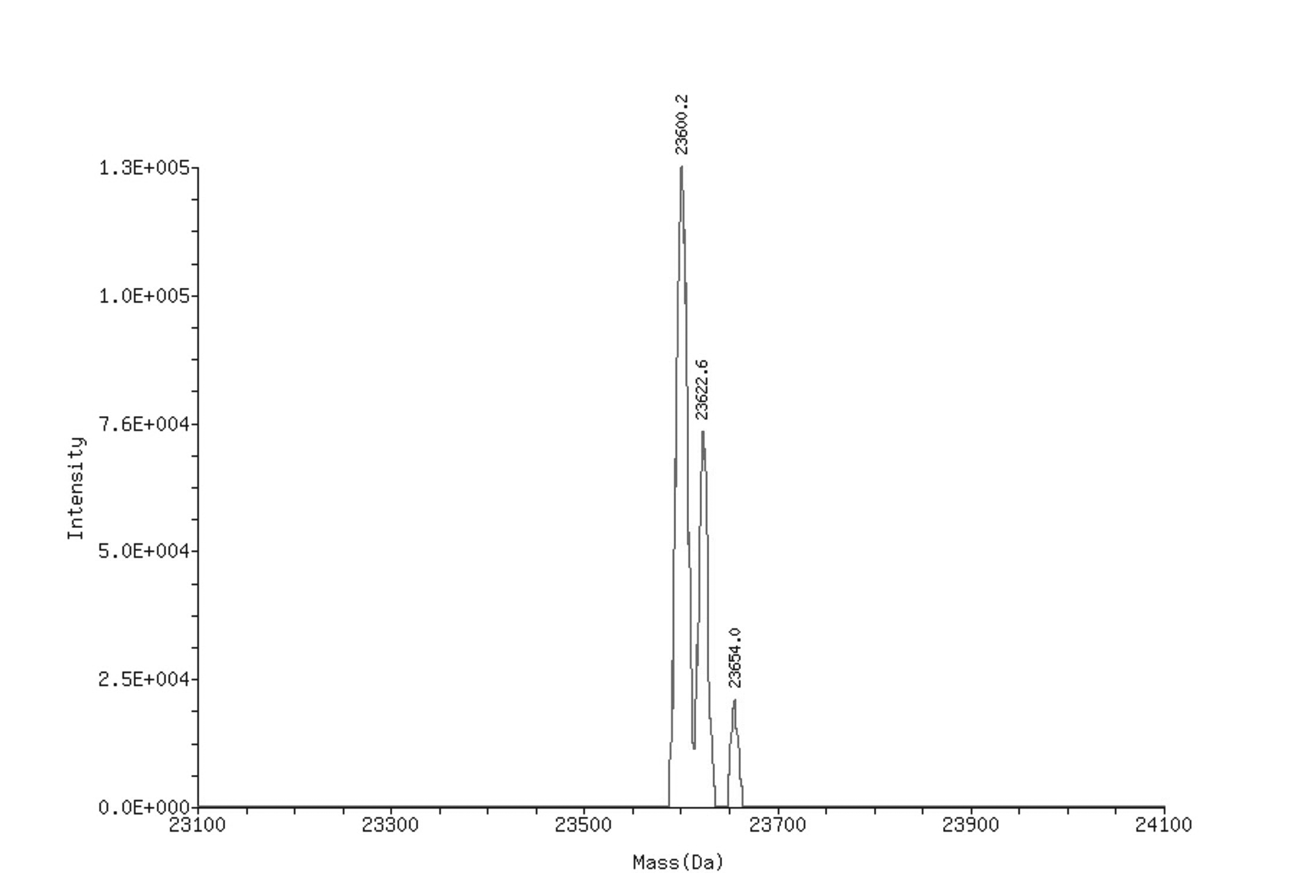

Supplement: FIG S1 [file sph003182577sf1.tif]

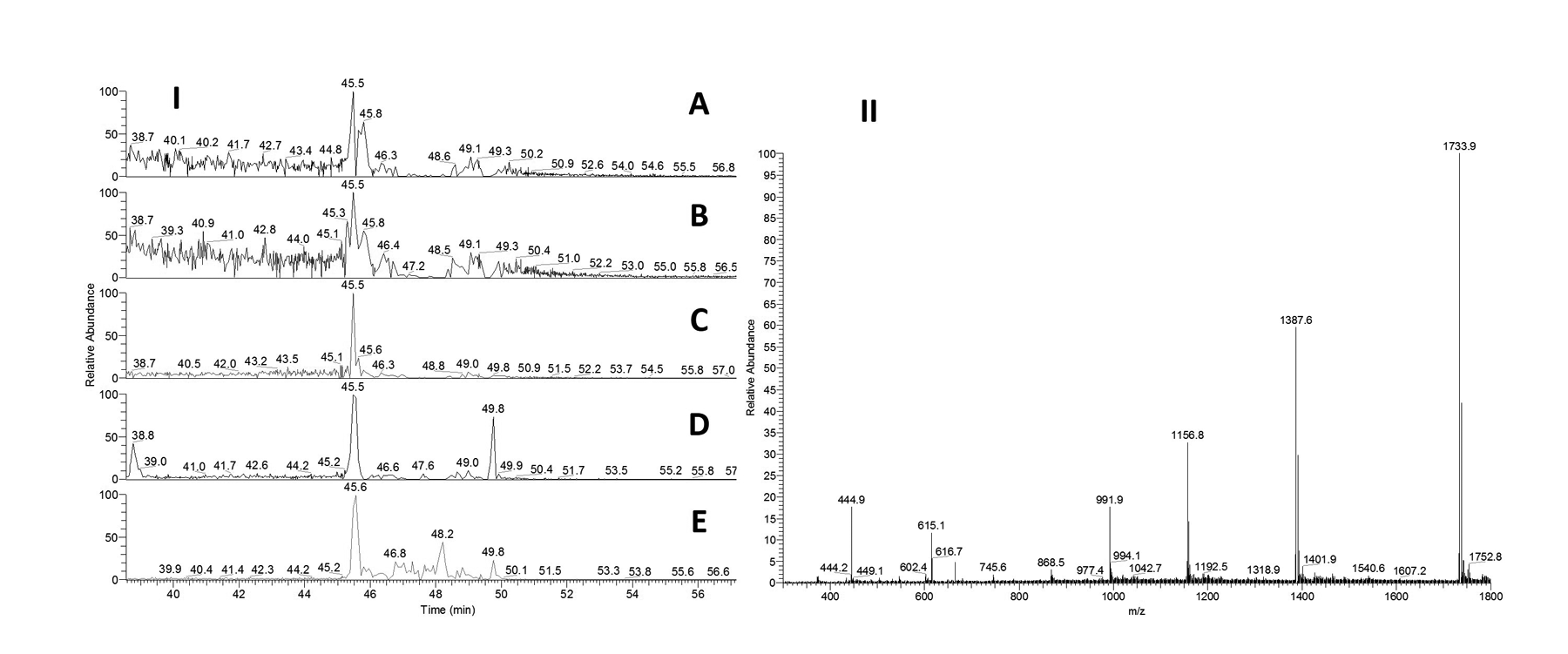

Supplement: FIG S2 [file sph003182577sf2.tif]

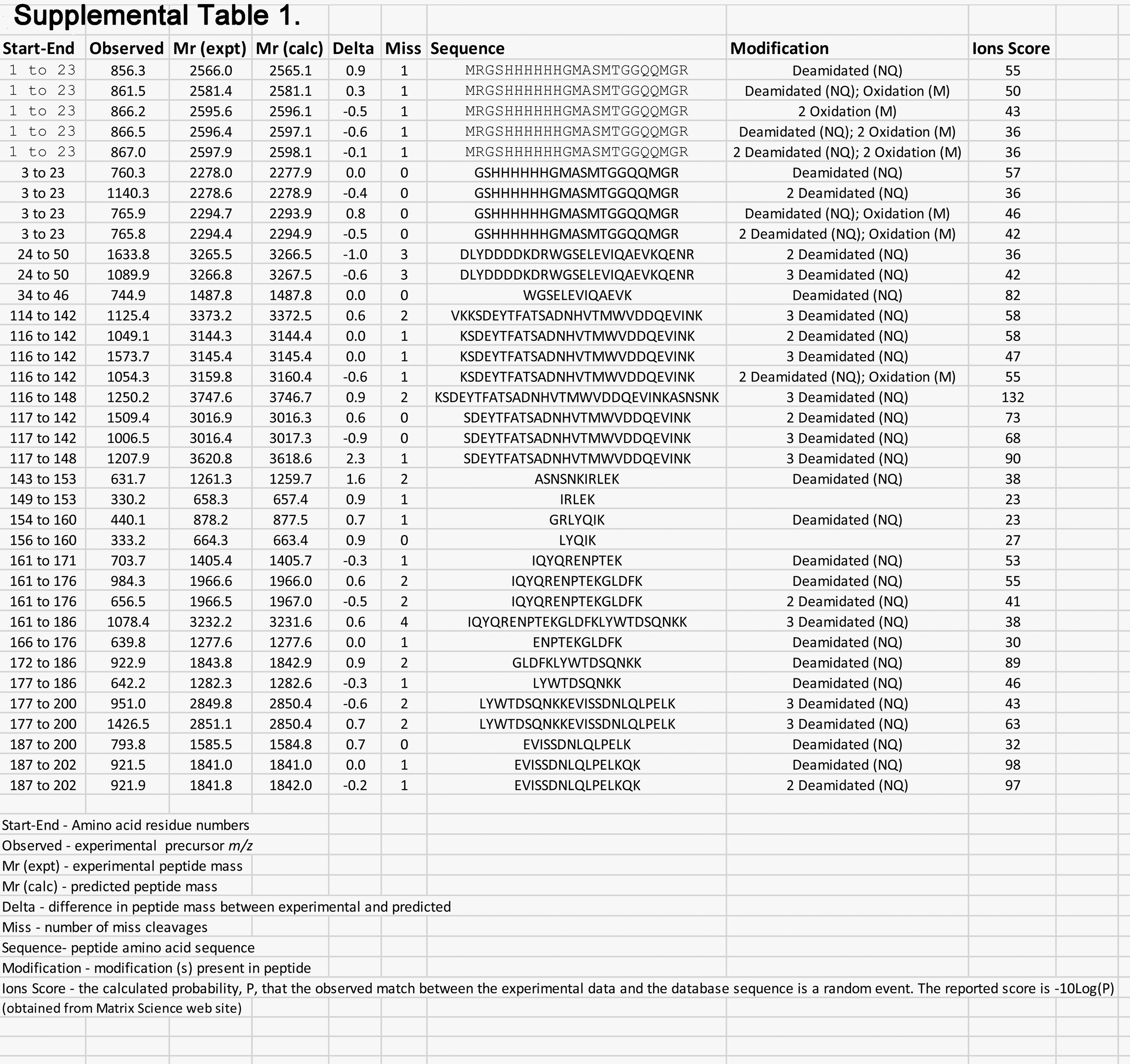

Supplement: TABLE S1 [file sph003182577st1.tif]
