# Supplementary material for: Serum-Mediated Cleavage of Bacillus anthracis Protective Antigen Is a Two-Step Process That Involves a Serum Carboxypeptidase
Source: mSphere. 2018 Jun 27;3(3):e00091-18. doi: 10.1128/mSphere.00091-18 (PMC6021598; doi:10.1128/mSphere.00091-18)
Supplement: TABLE S2 [file sph003182577st2.docx]

**Supplemental Table 2.**

| **Antibody** | **Isotype** | **PA Fragment** | **Specificity** |
| --- | --- | --- | --- |
| 7.5g | IgG_2b_ | PA_20_ | Domain 1 |
| 10F4 | IgG_1_ | PA_63_ | Domain 4 |
| 19D9 | IgG_1_ | PA_20_ | Domain1* |
| 20G7 | IgM | PA_20_ | Domain 1* |
| 2H9 | IgG_1_ | PA_63_ | Domains 2-4 |

* These antibodies compete with each other to bind Domain 1.
